# Using the question-behavior effect to change multiple health behaviors: An exploratory randomized controlled trial^☆^

**DOI:** 10.1016/j.jesp.2018.07.008

**Published:** 2019-03

**Authors:** Sarah Wilding, Mark Conner, Andrew Prestwich, Rebecca Lawton, Paschal Sheeran

**Affiliations:** a University of Leeds, UK; b University of North Carolina at Chapel Hill, United States of America

**Keywords:** Question-behavior effect, Health, Protection, Risk, Multiple behavior change

## Abstract

Asking questions about a behavior has been found to influence subsequent performance of that behavior, a phenomenon termed the *question-behavior effect* (QBE). The present study addressed two under-researched questions concerning the QBE: (1) Can the QBE be used to change multiple health behaviors, and (2) does enhancing dissonance during questionnaire completion increase the magnitude of the QBE? Participants (*N* = 1534) were randomized to one of three conditions (dissonance-enhanced QBE; standard QBE; control) that targeted three health-protective behaviors (eating fruit and vegetables, physical activity, dental flossing) and three health-risk behaviors (alcohol intake, sedentariness, unhealthy snacking). The dissonance-enhanced intervention comprised a message designed to pressurize participants into forming healthful behavioral intentions. Behavior was assessed via self-reports at four-week follow up. Findings showed significant overall effects of the QBE both in increasing performance of health-protective behaviors (*p* = .001) and in reducing performance of health-risk behaviors (*p* = .04). Compared to the standard QBE condition, the dissonance-enhanced QBE intervention increased performance of health-protective behaviors (*p* = .04) and marginally reduced performance of health-risk behaviors (*p* = .07). The dissonance-enhanced QBE intervention outperformed the control condition in all analyses. This is the first report that a brief QBE intervention influences performance of multiple health behaviors. Findings supported the idea that magnifying dissonance increases the impact of the QBE.

Health behaviors are associated with a range of long-term health outcomes (e.g., morbidity and mortality). For example, low rates of fruit and vegetable consumption and physical activity, together with high levels of alcohol consumption and smoking are estimated to account for two-thirds of cardiovascular disease, cancer, and all-cause mortality (Kvaavik, Batty, Ursin, Huxley, & Gale, 2010). Improvements in health outcomes thus depend not on merely changing one or two behaviors but on changing multiple health behaviors simultaneously (Wilson et al., 2015). Interventions that target multiple health behaviors are needed, and so the present research tested whether the question-behavior effect (QBE; Sprott et al., 2006) might be effective in that regard. The QBE reflects the fact that asking questions about a behavior changes subsequent performance of that behavior (e.g., Wood et al., 2016). Numerous studies have demonstrated the QBE for individual health behaviors (see review by Wilding et al., 2016), though the evidence is stronger for health-protective (e.g., fruit and vegetable consumption, physical activity) than for health-risk (e.g., alcohol consumption, smoking) behaviors. The present study tested the QBE in relation to a suite of six health behaviors (3 health-protective; 3 health-risk). Although likely to generate small effects, the QBE can be applied in simple, low-cost interventions as opposed to alternative, more costly interventions to address multiple health behavior change. The study also sought to test a proposed mechanism underlying the QBE (cognitive dissonance) by exploring a manipulation designed to increase the cognitive dissonance generated by completing questions about a behavior.

## 1 The question-behavior effect

The question-behavior effect refers to the finding that answering questions about a behavior produces a small-sized change in subsequent performance of that behavior (see Rodrigues, O'Brien, French, Glidewell, & Sniehotta, 2015; Spangenberg, Kareklas, Devezer, & Sprott, 2016; Wilding et al., 2016; Wood et al., 2016, for recent reviews). For example, Williams, Block, and Fitzsimons (2006) observed that asking students about their intentions to exercise increased subsequent self-reported exercise rates by 12% two months later, and Kypri, Langley, Saunders, and Cashell-Smith (2007) showed that asking questions about drinking levels reduced alcohol consumption by 18% twelve months later. A meta-analysis by Wood et al. (2016) reported that asking questions about intentions, multiple cognitions, satisfaction, self-predictions, and past behavior was associated with small but significant impacts on subsequent performance of the behavior (*d* = 0.24). In relation to health behaviors, Wilding et al. (2016) showed that the QBE had significantly stronger effects for promoting protective behaviors (e.g., taking physical activity) than for reducing risk behaviors (e.g., drinking alcohol), and recommended further studies testing both effects.

Lawrence and Ferguson (2012) undertook the only QBE study that tested the impact of assessing intentions and past behavior in relation to multiple health behaviors (quitting cigarette smoking, reducing alcohol use, practicing safe sex, driving safely, dieting, and exercising). Participants either completed a questionnaire tapping intention and past behavior in relation to each of the target behaviors, or no questionnaire, at baseline. Two months later, self-reported alcohol consumption was significantly lower for those completing compared to not completing a baseline questionnaire. None of the other behaviors were impacted significantly by the questionnaire. However, Lawrence and Ferguson's (2012) study did not use a Randomized Controlled Trial (RCT) design, recruited University students as participants, and was under-powered to detect the small sized effect commonly observed in QBE studies (Wilding et al., 2016; Wood et al., 2016). Further tests of the QBE in relation to multiple health behaviors are therefore warranted. The present study used a large and diverse sample and conducted an a priori power analysis based on the results of a recent review (Wilding et al., 2016). As Wilding et al. (2016) also found that risk of bias influenced effect sizes in QBE studies. The present study minimized risk of bias by recruiting an online sample that was blinded and allocation to condition was concealed; online recruitment also reduces potential experimenter demand effects (e.g., Birnbaum, 2004).

## 2 The role of dissonance in the question-behavior effect

One mechanism thought to underlie the QBE is cognitive dissonance (Spangenberg et al., 2016; Wood et al., 2016). The idea is that asking questions about a behavior activates personal or social norms and reminds people of any failures to meet those norms in the past (Dholakia, 2010). Dissonance is an aversive state and motivation to reduce this aversive state instigates change in subsequent behavior (Aronson, 1992). An implication of the cognitive dissonance explanation of the QBE is that the way the questions are asked must generate dissonance; the greater the dissonance generated by the questionnaire, the larger should be the QBE. Evidence indicates that cognitive dissonance is granular, and people can experience different degrees of dissonance (Leippe & Eisenstadt, 1999) that can be assessed with a ‘dissonance thermometer’ (Devine, Tauer, Barron, Elliot, & Vance, 1999).

We hypothesized that focusing the respondent's attention on the importance of health and behaving healthily would increase the extent of cognitive dissonance experienced while completing the questionnaire and enhance the magnitude of the QBE. That is, the present study attempted to augment dissonance in one condition (the dissonance-enhanced QBE condition) by highlighting the importance of health and behaving healthily as part of an introductory text presented at the beginning of the questionnaire. This manipulation was designed to powerfully activate norms about health behavior and make participants feel that they should both intend to behave healthily and act in line with their heightened intentions. Thus, the present study had three conditions: A control condition (no questions about target behaviors), a standard QBE condition (questions about target behaviors), and a dissonance-enhanced QBE condition (dissonance-inducing introductory text plus questions about target behaviors); effects were assessed on multiple health-protective and health-risk behaviors measured one month later. The present study offers the first attempt to directly manipulate dissonance in order to maximize the QBE, and thus offers a strong test of the cognitive dissonance explanation of the QBE (Spangenberg et al., 2016).

## 3 Aims and hypotheses

The present study tested (a) whether the QBE influences rates of performance of three health-protective and three health-risk behaviors, and (b) whether magnifying dissonance increases the behavioral impact of the QBE. We formed the following hypotheses:

1) Asking cognition and behavior questions about increasing health-protective behaviors will promote performance of these behaviors compared to the control condition;
2) Asking cognition and behavior questions about reducing health-risk behaviors will decrease performance of these behaviors compared to the control condition;
3) Magnifying dissonance will augment the QBE; participants in the dissonance-enhanced QBE condition will perform (a) health-protective behaviors more frequently, and (b) health-risk behaviors less frequently compared to either the standard QBE or control conditions.

## 4 Method

### 4.1 Participants

**Fig. 1:**
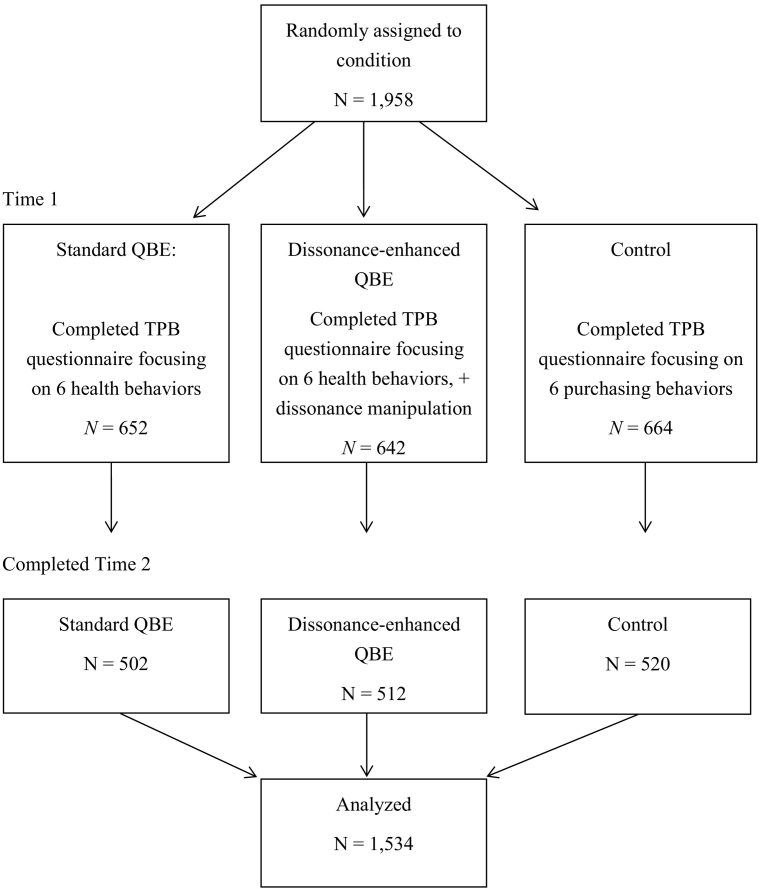
Participant flow diagram.

A priori sample size calculations using G*Power were conducted based on average effect sizes for QBE studies conducted for health behaviors and studies using an online setting (average *d* = 0.17; Wilding et al., 2016). This indicated that with this small sized effect, an alpha of *p* = .05 and 80% power, a total of 1338 participants spread across three conditions was required. Participants were recruited via Prolific Academic, an online database of participants interested in taking part in research from a range of academic areas. In order to allow for drop out we aimed to recruit 2000 participants at baseline (i.e., the study was capped at a maximum of 2000 participants). From a database of approximately 26,000 potential participants, a total of 1958 individuals were recruited and completed part 1 of the study. Fig. 1 shows the recruitment flow diagram. A total of 1534 participants subsequently completed the four-week follow-up and could be matched to time 1 data. Data were collected between June and August 2016. Participants were paid £4.30 ($6.03 at the time of recruitment) for completing both parts of the study. Table 1 shows the demographic characteristics for these 1534 participants.

**Table 1:** Demographic characteristics and effects on behavior at follow-up by condition.

	Standard QBE		Dissonance-enhanced QBE		Control group	
	*N* = 502		*N* = 512		*N* = 520	
Variables^1^	*N*	*M* (or %)	*N*	*M* (or %)	*n*	*M* (or %)
Demographic characteristics^1^						
Age	502	32.6	512	31.3	520	31.4
Gender (%)						
Male	244	48.6	238	46.5	272	52.3
Female	251	50.0	268	52.3	243	46.7
Non-binary	7	1.4	6	1.2	5	1.0
Ethnicity (%)						
Non-Caucasian	130	25.9	149	29.1	152	29.2
Caucasian	372	74.1	363	70.9	368	70.8
Education (%)						
Below undergraduate degree	208	41.4	224	43.8	205	39.4
Undergraduate degree/above	294	58.6	288	56.3	315	60.6
Employment (%)						
Full/part time employment	328	65.3	324	63.3	338	65.0
Full/part time student	90	17.9	104	20.3	93	17.9
Not currently working	84	16.7	84	16.4	89	17.1
Effects on behavior						
Health-risk behaviors		0.01 (0.47)^ab^		−0.04 (0.48)^a^		0.04 (0.51)^b^
Health-protective behaviors		−0.003 (0.63)^b^		0.08 (0.63)^a^		−0.08 (0.62)^b^

*Note*. Means with different superscripts differ significantly at *p* < .05. The difference between the QBE-only and QBE plus dissonance condition is marginally significant (*p* = .07) for health-risk behaviors.

1 There were no significant differences between conditions on demographic variables.

### 4.2 Procedure

The study was approved by a University Ethics Committee. Participants first gave informed consent and were then randomized to complete one of six questionnaires (3 conditions × 2 orders of questions). (Conditions are described below.) As questionnaire order had no significant effects and did not interact with condition, this factor is not considered further. Next, participants provided their demographic details before completing the questionnaire relevant to their assigned condition. After four weeks, participants reported their performance of six health behaviors over the previous four weeks. Participants also completed personality scales (after completing the behavior measures) that are not relevant to the present report. Finally, all participants were fully debriefed, thanked, and paid.

### 4.3 Design

Participants were randomized to one of three conditions using a random number generator when participants clicked on the study link: (1) Standard QBE condition (questionnaire about health behaviors), (2) Dissonance-enhanced QBE condition (health behavior questionnaire plus manipulation described below), (3) Control condition (questionnaire related to purchasing behavior). Participants and researchers were blinded to condition. The dependent variable was self-reported performance of each of the six health behaviors during the past month at one-month follow-up. All purchase behaviors questioned in the control condition were selected to reduce confounding with health behavior (i.e., they did not encourage specific food purchases, physical activities, or sedentary behavior).

The questionnaire order was counterbalanced so that participants in the experimental conditions (standard QBE or dissonance-enhanced QBE) were randomly allocated to either receive the questions on health-protective behaviors first or were allocated to receive the health-risk questions first.

All respondents first completed demographic questions tapping gender, age (split into <30 versus ≥30 years of age), ethnicity (split into Caucasian versus non-Caucasian), education (split into completed higher education versus not), and employment (split into student, employed, or not employed). Nationality, income and two ladder measures of socioeconomic status (Adler, Epel, Castellazzo, & Ickovics, 2000) were also assessed but were not further analyzed here.

### 4.4 Health behavior questionnaire

Participants in the two QBE conditions received a health behavior questionnaire. There were three health-protective behaviors (eating fruit and vegetables, performing recommended levels of physical activity, flossing daily), and three health-risk behaviors (not drinking over recommended levels per week, not sitting for extended periods of time, not consuming unhealthy snacks). The questionnaire comprised a total of 102 items, 15 cognition items for each of the six behaviors plus two past behavior items for each of the six behaviors presented at the end of the questionnaire. Each behavior was introduced and defined at the start of the set of questions. Questions were based on guidance for developing Theory of Planned Behavior questionnaires (Conner & Sparks, 2015) and asked participants to complete a 7-point Likert scale relating to performing behavior over the next four weeks. All questions were anchored from ‘Strongly agree-Strongly disagree’ unless otherwise stated.

Cognitions were assessed for each behavior as follows: Five intention items (e.g. “I am likely to”, “I intend to”, “I want to”, “I feel I should”, “I expect to, …eat five portions of fruit and vegetables per day over the next four weeks”); one self-efficacy item (“If it were entirely up to me, I am confident that I could…”); one perceived behavioral control item (“How much control do you believe you have over…” No control-complete control); four attitude items (“Eating five fruit and vegetables a day over the next four weeks would be”… Worthwhile-Worthless; Not enjoyable-Enjoyable; Important-Unimportant; Unpleasant-Pleasant); one injunctive norm item (“Most people important to me think that” I should-I should not …eat five fruit or vegetables a day over the next four weeks); one descriptive norm item (e.g. “I think that most people who are important to meat five fruit and vegetables a day”); one prioritization item (“I would prioritize … over other goals important to me”); one context stability item (e.g., “Is … something that you would do at the same times and in the same places each time?”, definitely no-definitely yes); and two past behavior items (“…is not something I do or plan to do”; “How often do you …?”, Never-always). Items were scored such that higher scores indicated a more positive view of the healthier behavior. The full set of items is available from the corresponding author.

### 4.5 Dissonance-enhancing manipulation

After completing demographic details and before completing the health behavior questionnaire, participants in the dissonance-enhanced QBE condition were exposed to a message that emphasized the importance of healthy living and described how much behavior determines one's health. The text explained that up to 1/3 of cancers and 80% of heart disease, stroke and Type 2 diabetes could be prevented if smoking, unhealthy diet, lack of physical activity, and alcohol use were removed as risk factors. The message also emphasized the importance of behaving healthily and encouraged participants to “Make the healthy choice!” The goal of the message was to induce participants to form more healthful intentions than they otherwise might, and evoke dissonance should they fail to realize those intentions.

As dissonance is notoriously difficult to measure directly (e.g., Aronson, 1992), we adopted an indirect approach that relied on participants' sense of feeling pressured to give answers that favored their health (see Sheeran et al., 2017). In particular, participants in the dissonance-enhanced QBE condition answered two questions about experiencing pressure to give favorable answers in relation to each of the six health behaviors (“I gave answers to the survey questions that I thought I should give, rather than what I really believe about…” and “The answers that I have given to the survey questions were more positive than my real views about…”; 7-point scales, ‘Definitely no-Definitely yes’; *r*s = 0.58 to 0.64). We anticipated that scores on this measure would be correlated with how strongly participants intended to perform health-protective behaviors and reduce health-risk behaviors.^1^^,^^2^

### 4.6 Purchase behavior questionnaire

Participants in the control condition completed equivalent items (15 cognition items plus two past behavior items for each of six behaviors) as participants in the experimental conditions but the items related to six purchase behaviors: purchasing groceries, purchasing toiletries and/or cosmetics, purchasing household cleaning items, reducing clothing purchasing, reducing music purchasing (including digital downloads), and reducing spending.

### 4.7 Outcome measures

At 4-week follow-up, all participants were asked three questions about their frequency of performance of each the six health behaviors (“How often did you eat at least five portions of fruit or vegetables each day?” Never, rarely, sometimes, often, always; “On how many days did you eat 5 portions of fruit and vegetables over the past four weeks?”; “Over the past four weeks I ate at least 5 portions of fruit and vegetables per day” Strongly disagree – Strongly agree). The three measures for each behavior were standardized and averaged to form the measure of behavior at follow-up (αs = 0.56–0.95). These were the primary outcome measures.

After completing the behavior measures, participants also completed a single item tapping intentions to perform each behavior (e.g., “I intend to eat at least five fruit and vegetables a day over the next four weeks”); a thirteen item self-monitoring scale (Lennox & Wolfe, 1984), a ten item conscientiousness scale (taken from the International Personality Inventory Pool; ipip.ori.org) and the 21-item personal life investments schedule (Staudinger & Fleeson, 1996). None of these measures were further analyzed here.

### 4.8 Analyses

Representativeness and randomization checks used Chi-square and one-way ANOVA to test for differences on demographic variables. The manipulation check used a mixed-model ANOVA and correlations. The analyses for behavior at follow-up involved a mixed-model hierarchical ANOVA with one overarching factor (QBE: Experimental vs. control) and a nested factor representing the Type of QBE (dissonance-enhanced QBE vs. standard QBE), with type of behavior (protective vs. risk) as a within-subjects factor. Interactions with type of behavior were decomposed by examining risk and protective behaviors separately and included specific behavior (3 levels representing the three health risk behaviors or three health protection behaviors) as a within-subjects factor.

## 5 Results

### 5.1 Randomization and representativeness checks

Randomization to condition was successful. There were no significant differences between the three conditions for gender *χ*^2^(2, *N* = 1958) = 4.79, *p* = .09, ethnicity *χ*^2^(2, *N* = 1958) = 3.12, *p* = .21, education *χ*^2^(2, *N* = 1958) = 2.06, *p* = .36, age group *χ*^2^(2, *N* = 1958) = 4.08, *p* = .13, or employment status *χ*^2^(4, *N* = 1958) = 2.43, *p* = .66.

There were significant differences between participants who completed the baseline questionnaire only as compared to participants who completed both the baseline and follow-up questionnaires. Participants who completed both questionnaires were more likely to be in the older age group, *χ*^2^(1, *N* = 1958) = 4.50, *p* = .03, and were more likely to have completed higher education, *χ* ^2^(1, *N* = 1958) = 12.82, *p* < .001. There were no differences for gender *χ*^2^(1, *N* = 1958) = 1.60, *p* = .21, ethnicity *χ*^2^(1, *N* = 1958) = 0.56, *p* = .45, or employment status *χ*^2^(2, *N* = 1958) = 4.60, *p* = .10.

### 5.2 Manipulation checks for dissonance intervention

Two sets of analyses were used to check whether the dissonance manipulation was successful. First, we anticipated that participants in the dissonance-enhanced QBE condition would have higher intentions to perform health-protective behaviors and to reduce health-risk behaviors compared to the standard QBE condition. A 2 (Type of QBE condition: standard QBE vs. dissonance-enhanced QBE) × 6 (Type of behavior) mixed-model ANOVA revealed a significant main effect of Type of QBE condition, *F*(1, 1292) = 11.41, *p* < .01, eta^2^ = 0.009. As predicted, intentions were significantly higher in the dissonance-enhanced QBE condition (*M* = 4.75, *SD* = 1.05) than the standard QBE condition (*M* = 4.55, *SD* = 1.07). There was a significant effect of behavior, *F*(5, 6460) = 172.46, *p* < .01, eta^2^ = *0*.118. However, there was no significant interaction between Type of QBE condition and Type of behavior, *F*(5, 6460) = 0.95, *p* = .45, eta^2^ = *0.001*, indicating that the increase in intention scores was equivalent for all six health behaviors.

Second, we anticipated that the dissonance-enhanced QBE condition would exhibit increased intention scores precisely because the manipulation made participants feel that they ought to rate their intentions as higher. Consistent with this idea, there was a significant correlation between scores on the dissonance items and intention ratings (*r* = 0.22, *p* < .001) in the dissonance-enhanced QBE condition; higher dissonance scores were associated with stronger intentions to perform healthier behaviors. Thus, the dissonance-enhanced QBE intervention passed both manipulation checks.

### 5.3 Impact of the QBE conditions on behavior at follow-up

**Fig. 2:**
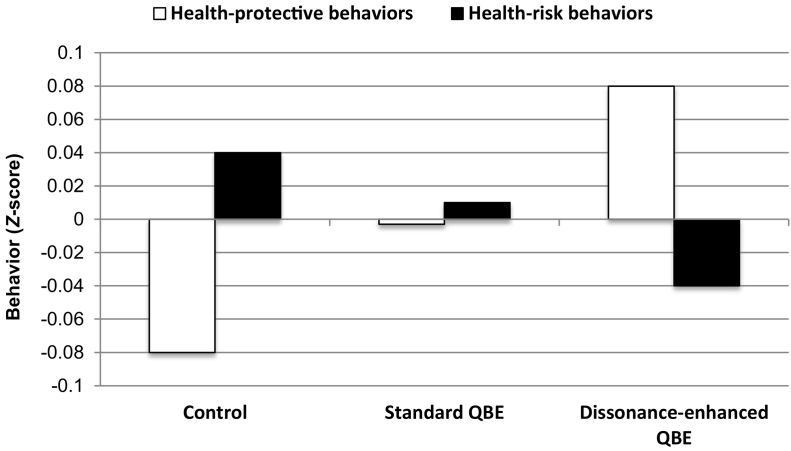
Health-protective and health-risk behaviors at follow-up by condition.

The results of the hierarchical mixed-model ANOVA showed a significant overall effect of the QBE, *F*(3, 1529) = 4.63, *p* = .003, eta^2^ = *0.009*. No nested effect of the Type of QBE condition (dissonance-enhanced QBE vs. standard QBE) was observed, *F*(3, 1529) = 0.72, *p* = .54, eta^2^ = *0.001*. However, there was a significant interaction between type of behavior (protective vs. risk behavior) and condition (QBE vs. control), *F*(3, 1529) = 3.87, *p* = .009, eta^2^ = *0*.008. We decomposed the interaction by conducting separate analyses for health-risk and health-protective behaviors (see Table 1; Fig. 2).

#### 5.3.1 Risk behaviors

There was a significant main effect of the overarching QBE vs. control factor indicating lower frequency of performing risk behaviors in the QBE conditions than the control condition (QBE-*M* = −0.01, *SD* = 0.48; Control-*M* = 0.04, *SD* = 0.51), *F*(1, 1531) = 4.08, *p* = .04, eta^2^ = *0.003*. There was a marginally significant effect of the nested factor, Type of QBE condition, *F*(1, 1531) = 3.39, *p* = .07, eta^2^ = 0.002. Pairwise comparisons again indicated that performance of health-risk behaviors was significantly (*p* = .008) higher in the control condition compared to the dissonance-enhanced QBE condition, but was not significantly different between the control and the standard QBE condition (*p* = .41). Risk behaviors were performed marginally significantly (*p* = .07) less frequently by participants in the dissonance-enhanced QBE condition than the standard QBE condition. Thus, the overall significant effect of the QBE factor was due to the dissonance-enhanced QBE condition. There was no behavior × QBE interaction, *F*(2, 3062) = 1.10, *p* = .33, eta^2^ = *0*.001 suggesting this QBE effect did not vary across the three different risk behaviors.

#### 5.3.2 Protective behaviors

There was a significant main effect of the QBE indicating greater frequency of performance of health-protective behaviors in the QBE conditions compared to the control condition (QBE-*M* = 0.04, *SD* = 0.62; Control-*M* = −0.08, *SD* = 0.63), *F*(1, 1530) = 11.02, *p* = .001, eta^2^ = *0.007*. There was also a significant effect of the nested factor, Type of QBE condition, *F*(1, 1531) = 4.22, *p* = .04, eta^2^ = 0.003. Pairwise comparisons again indicated that performance of health-protective behaviors was significantly (*p* < .001) lower in the control condition compared to the dissonance-enhanced QBE condition, but was not significantly different between the control and the standard QBE condition (*p* = .07). Protective behaviors were also performed significantly (*p* = .04) more frequently by participants in the dissonance-enhanced QBE condition than the standard QBE condition. There was no behavior **×** QBE interaction, *F*(2, 3062) = 1.05, *p* = .35, eta^2^ = 0.001 indicating that all three protective behaviors increased as a result of exposure to the dissonance-enhanced QBE condition.

## 6 Discussion

The *question-behavior effect* (QBE) has a small effect (Cohen, 1988) in promoting health behaviors (e.g., *d* ≈ 0.14; Wilding et al., 2016). However, even such small effects of brief, low-cost interventions can be important when interventions are applied at a population level (e.g., Babor et al., 2007; Wutzke, Shiell, Gomel, & Conigrave, 2001), especially if the intervention can change multiple health behaviors and is scalable (Kazdin & Blasé, 2011). The intervention tested here meets these criteria and therefore could represent a useful way to help change health behaviors and have an impact on population-level health. The present research offers two advances in using the QBE to promote health behaviors: (1) This is the first study to test the impact of the QBE on multiple health behaviors – including both health-risk and health-protective behaviors – among a large sample using an RCT design; and (2) We tested a novel dissonance-enhanced QBE condition and compared its behavioral impact both to a standard QBE intervention and a control condition (a questionnaire that did not concern health behaviors). The key findings were that the QBE proved effective both at increasing performance of health-protection behaviors and in reducing performance of health-risk behaviors. There was also evidence that magnifying dissonance (dissonance-enhanced QBE condition) enhanced effects on health behaviors beyond that engendered by a standard QBE intervention.

Supporting Hypothesis 1, the QBE generated a significant increase in protection behaviors during the follow-up period. Of interest, however, pairwise comparisons indicated that this effect was due to the dissonance-enhanced QBE condition; the standard QBE condition did not increase performance compared to the control condition. Supporting Hypothesis 2, there was an overall reduction in health-risk behaviors due to the QBE. Again, we observed that the overall QBE was driven by the dissonance plus QBE condition; the pairwise difference between the standard QBE and control conditions was not significant. The present findings can thus be seen to offer support both for advocates (e.g., Wilding et al., 2016; Wood et al., 2016) and critics (e.g., Rodrigues et al., 2015; van Dongen, Abraham, Ruiter, & Veldhuizen, 2013) of the use of the QBE to promote health behaviors. On the one hand, we observed significant main effects for the combined QBE conditions and for the dissonance-enhanced QBE condition compared to the control condition for both health-risk and health-protection behaviors. On the other hand, the standard QBE intervention did not generate significant improvements in these behaviors compared to the control condition. Thus, the present findings may suggest caution in using standard QBE interventions to change multiple behaviors but also indicate that QBE interventions that magnify dissonance can be effective in this regard.

The fact that the dissonance-enhanced QBE condition increased health-protective behaviors and reduced health-risk behaviors compared to the control condition whereas the standard QBE did not, offers one line of evidence supporting the superiority of the dissonance-enhanced QBE intervention tested here. Comparisons of the dissonance-enhanced QBE and standard QBE conditions also broadly supported this conclusion. Performance of health-protection behaviors was significantly higher (*p* = .04) in the dissonance-enhanced QBE condition and performance of health-risk behavior was marginally (*p* = .07) lower. These findings suggest that the magnitude of the QBE can be enhanced by deploying messages designed to evoke dissonance by highlighting potential discrepancies between intentions and health actions. We observed that a dissonance-based message increased behavioral intentions, and led to concomitant changes in subsequent health behaviors. In terms of health significance and potential applicability of these findings, the results support a small effect of the QBE on multiple behaviors. While the effects on behavior were small, the brief nature of the intervention and its online delivery method support its potential wide reach.

The present findings also support the idea that cognitive dissonance is a key mechanism underlying the QBE. A manipulation designed to maximize cognitive dissonance led to stronger behavioral impacts of the QBE. This finding is important because although reviews of the QBE have tended to find more support for the cognitive dissonance than other potential mechanisms (Spangenberg et al., 2016; Wood et al., 2016), the evidence has tended to be rather indirect and less than conclusive. Our finding that a manipulation designed to increase the amount of cognitive dissonance experienced while completing questions about a behavior provides firmer support for cognitive dissonance as the mechanism underlying the QBE. We acknowledge that the amount of cognitive dissonance generated in the standard QBE condition was not assessed, which precluded direct comparison of the standard QBE and dissonance-enhanced QBE conditions. Direct comparison of dissonance generated in different QBE conditions should be a priority in future studies. In addition, future QBE studies might usefully explore when dissonance promotes behavior as opposed to attitude change.

The present research has both strengths and limitations. On the plus side, we used a RCT design among a large and diverse sample that was powered *a priori* to detect a small-sized QBE (e.g., Wilding et al., 2016), and involved a four-week follow-up. Recent reviews of the QBE literature (Rodrigues et al., 2015; Wilding et al., 2016) observed both a high risk of bias in previous tests of the QBE and larger effects in higher-risk as compared to lower-risk studies. The present study was explicitly designed to reduce potential for risk of bias. Nonetheless, there are several limitations of the present study that should be acknowledged. First, behavior at follow-up relied upon self-report, and a stronger test would have been afforded by objective measures of behavior. However, the QBE has been demonstrated to influence both self-reported and objective measures of behavior (e.g., Godin, Sheeran, Conner, & Germain, 2008) and type of behavior measure (self-report vs. objective) did not moderate the magnitude of the QBE in recent reviews (Wilding et al., 2016; Wood et al., 2016). A second potential problem concerns participants' expectancies. Perhaps participants guessed that the researchers wanted them to undertake the relevant health behaviors and merely acceded to that perceived demand. To test this idea, we recruited new participants through Prolific Academic (*N* = 102) who were equivalent to the sample used here in terms of in gender, ethnicity, and education. Participants were asked whether they believed that completing surveys on protective or risk behaviors would influence their behavior. Given that there are four possible answers (yes to both questions; no to risk, yes to protective, yes to protective, no to risk; no to both questions), the prior probability of the correct answer (yes to both) is 25%. In fact, 22% of participants got the correct answer which is not significantly different from 25%. Thus, it seems unlikely that participants' expectancies can account for the present findings.

Third, participants who completed both waves of data collection were older and better educated than participants who completed the first wave only. Caution is therefore warranted in making generalizations to other samples. Relatedly, while the use of an online sample is justified in this, the first strong test of the impact of the QBE on multiple behaviors, we acknowledge that further tests using other recruitment strategies are needed in order to better understand the applicability of the QBE. Fourth, completing the questionnaire in the dissonance-enhanced QBE condition may have taken longer than was the case in the standard QBE and control conditions, which could potentially explain the superior impact of this condition. Although we did not measure the time taken in the different conditions, this explanation seems unlikely as the dissonance-enhanced QBE condition only required the reading of a short piece of text plus answering two additional questions compared to the other two conditions. Finally, the study involved just six health behaviors and a 4-week follow-up period. Longer term follow-ups using a greater range of health behaviors would be desirable in order to assess the generality and durability of the QBE on behavior change.

Finally, although the present research focused on multiple behavior change, we were unable, in a single study, to address several contextual factors that might influence the strength of the QBE. For instance, the setting in which questions are asked and the mode of question delivery could both be influential. Wilding et al. (2016) observed stronger QBEs in the laboratory compared to other settings and using face-to-face delivery compared to other modes (e.g., mail, phone, internet). Similarly, the context of performance of the respective behaviors could strengthen or weaken the impact of questioning. It is not yet clear whether the QBE is similarly effective for behaviors that differ in terms of their frequency of performance and the stability of their context of performance (nonhabitual and habitual behaviors; Ouellette & Wood, 1998). Relatedly, a fine grained analysis remains to be undertaken to discover what contexts are especially liable to evoke the QBE *in situ* and in what contexts does behavior change not occur even though participants were questioned about their future behavior. These shortcomings of the present study suggest valuable directions for future research in exploring how different contextual features might moderate the QBE.

Notwithstanding these limitations, the present study advances understanding of the QBE as a technique for promoting health behavior change. Whereas previous research focused on single health actions (e.g., screening, vaccination, blood donation, physical activity), the current study offers new evidence that the QBE can be used to change multiple health behaviors, and can both increase performance of health-protective behaviors and reduce performance of health-risk behaviors. The present study also indicates that standard QBE interventions are not always effective but that effectiveness can be enhanced by using messages designed to enhance dissonance. Based on these findings, further tests of dissonance-enhanced, QBE interventions targeting multiple health behaviors would seem warranted.

## Footnotes

1 The design specified that questions about pressure be asked in both the Dissonance-enhanced QBE and the standard QBEconditions so that scores could be compared between conditions. However, a programming error resulted in these dissonance check items appearing only in the dissonance-enhanced QBE condition. For this reason, the best evidence that we can present about the success of the dissonance manipulation is that (a) intentions are significantly healthier in the dissonance-enhanced QBE condition compared to the standard QBE condition, and (b) there is a significant correlation between the dissonance check and intentions scores in the dissonance-enhanced QBE condition.

2 There is independent evidence that the present manipulation indeed evokes dissonance. Drawing upon Steele and Liu's (1983) demonstration that affirming a valued aspect of the self can eliminate dissonance and its accompanying cognitive changes, Sheeran et al. (2017) observed that a self-affirmation manipulation abolished the relationship between the dissonance check items used here and subsequent behavior. That is, self-reported experience of pressure was associated with behavior change in the circumstances operating in the present study, but was no longer related to behavior when participants self-affirmed (and dissonance was thereby eliminated).
